# Effects of green tea polyphenol on methylation status of *RECK* gene and cancer cell invasion in oral squamous cell carcinoma cells

**DOI:** 10.1038/sj.bjc.6604521

**Published:** 2008-07-29

**Authors:** K Kato, N K Long, H Makita, M Toida, T Yamashita, D Hatakeyama, A Hara, H Mori, T Shibata

**Affiliations:** 1 Department of Oral and Maxillofacial Sciences, Gifu University, Graduate School of Medicine, 1-1 Yanagido, Gifu 501-1194, Japan; 2 Department of Tumor Pathology, Gifu University, Graduate School of Medicine, Gifu 501-1194, Japan

**Keywords:** green tea polyphenol, EGCG, *RECK* hypermethylation, oral cancer invasion

## Abstract

*RECK* is a novel tumour suppressor gene that negatively regulates matrix metalloproteinases (MMPs) and inhibits tumour invasion, angiogenesis and metastasis. In the present study, we investigated the effects of epigallocatechin-3-gallate (EGCG), a major polyphenol in green tea, on the methylation status of the *RECK* gene and cancer invasion in oral squamous cell carcinoma cell lines. Our results showed that treatment of oral cancer cells with EGCG partially reversed the hypermethylation status of the *RECK* gene and significantly enhanced the expression level of *RECK* mRNA. Inhibition of MMP-2 and MMP-9 levels was also observed in these cells after treatment with EGCG. Interestingly, EGCG significantly suppressed cancer cell-invasive ability by decreasing the number of invasive foci (*P*<0.0001) as well as invasion depth (*P*<0.005) in three-dimensional collagen invasion model. Although further investigation is required to assess the extent of contribution of *RECK* on MMPs to the suppression of invasive behaviour, these results support the conclusion that EGCG plays a key role in suppressing cell invasion through multiple mechanisms, possibly by demethylation effect on MMP inhibitors such as *RECK*.

Cancer development is a multistage process requiring progressive genetic and epigenetic changes in neoplastic and responding stromal cells. During the process of malignant progression, migration of cells into the underlying extracellular matrices is a fundamental feature of tumour invasion. The reversion-inducing cysteine-rich protein with Kazal motifs (RECK), a novel matrix metalloproteinases (MMPs) inhibitor, was originally isolated as a transformation suppressor gene against activated *ras* oncogenes (Takahashi *et al*, 1998; Oh *et al*, 2001; Sasahara *et al*, 2002; Noda *et al*, 2003; Simizu *et al*, 2005; Chang *et al*, 2006). Previous studies have revealed that *RECK* is able to inhibit tumour angiogenesis, invasion, and metastasis. Its downregulation has been shown in several types of human cancers. Recently, the decrease in *RECK* expression is reported to correlate with hypermethylation of the promoter region (Furumoto *et al*, 2000; Song *et al*, 2006; Chang *et al*, 2007; Cho *et al*, 2007). In human cancers, aberrant methylation of tumour suppressor genes is of comparable significance to classic genetic mutations (Lund and van Lohuizen, 2004; Ha and Califano, 2006; Stresemann *et al*, 2006; Shaw, 2006). The study of the patterns of gene silencing because of hypermethylation would therefore help to understand and predict cancer cell behaviour and responsiveness to various treatments of cancers (Hellebrekers *et al*, 2007; Shaw *et al*, 2007).

Tea is the most widely consumed beverage worldwide. Tea components, especially green and black tea constituents, have been reported to prevent carcinogenesis *in vitro* and *in vivo* (Jung and Ellis, 2000; Benelli *et al*, 2002; Yang *et al*, 2002; Chung *et al*, 2003; Fang *et al*, 2003; Fassina *et al*, 2004; Ju *et al*, 2005; Khan *et al*, 2006). Epigallocatechin-3-gallate (EGCG), the major polyphenol in green tea, is believed to be a key active ingredient. Previous studies have shown that EGCG is methylated by catechol-*O*-methyltransferase and inhibits DNA methyltransferase (DNMT). The inhibition of DNMT would block the hypermethylation of the newly synthesised DNA strand, resulting in the reversal of the hypermethylation and the re-expression of the silenced genes (Fang *et al*, 2003; Lund and van Lohuizen, 2004; Ha and Califano, 2006; Stresemann *et al*, 2006; Shaw, 2006; Hellebrekers *et al*, 2007; Shaw *et al*, 2007). Other DNMT inhibitors such as 5-aza-2′-deoxycytidine (5-aza-dC) and zebularine also have similar inhibitory effect. Although there is a high potential for developing this group of inhibitors for cancer therapy, side effects and toxicity are serious concerns. The chemoprevention of cancer by tea components as natural inhibitors of DNMT is therefore such a promising approach with less side effects and toxicity (Chung *et al*, 2003; Lambert and Yang, 2003; Szyf *et al*, 2004; Ju *et al*, 2005; Wilson *et al*, 2007).

In the present study, we determined the effects of EGCG treatment on the methylation status and expression level of the *RECK* gene in human oral squamous cell carcinoma cell lines. The inhibition of oral carcinoma invasion by EGCG was also examined by a three-dimensional collagen invasion model.

## Materials and methods

### Cell lines and cell cultures

Four human oral squamous cell carcinoma cell lines HSC3, HSC4, SCC9, SCC25 and human cervical cancer cell line HeLa were examined. These cell lines were obtained from Cell Resource Center for Biomedical Research (Tohoku University, Sendai City, Japan). All cell lines were maintained in RPMI-1640 (Sigma-Aldrich Company, St. Louis, MO, USA) supplemented with 10% fetal bovine serum (FBS) (Life Technologies Inc., Gaithersburg, MD, USA) and 50 000 U penicillin, 50 mg streptomycin at 37°C in a 5% CO_2_ humidified atmosphere. The cancer cell lines were also cultured in medium with 50 *μ*M EGCG (Wako Pure Chemical Industries, Ltd., Osaka, Japan) or 8.7 *μ*M 5-aza-dC (MP Biomedicals, LLC, Eschwege City, Germany) for 6 days and harvested for further analyses as previously described (Fang *et al*, 2003). To determine the dose-dependent changes, SCC9 and HSC3 cell lines were treated with 5, 10, 20, or 50 *μ*M of EGCG or 8.7 *μ*M of 5-aza-dC for 6 days. EGCG or 5-aza-dC was added, in new culture medium, to the cells on days 1, 3, and 5. For the time course study, the cells were treated with 50 *μ*M of EGCG for 36, 72, or 144 h.

### Bisulphite modification and methylation-specific PCR

One microgram of the purified DNA was subjected to bisulphite modification. Bisulphite modification was performed using CpGenome™ DNA Modification Kit (Chemicon International, Temecula, CA, USA) according to the manufacturer's instructions. For MSP, the following primer sets were used: for methylated DNA, MF_RECK (5′-GTTAGTTTTTTTTTTTATTTTAGTGGTTCGA-3′) and MR_RECK (5′-TCCAAAACCTCCCGAAAACGAAAACG-3′), and for unmethylated DNA, UF_RECK (5′-GGTTAGTTTTTTTTTTTATTTTAGTGGTTTGA-3′) and UR_RECK (5′-ATTTCCAAAACCTCCCAAAAACAAAAACA-3′). Reactions were performed in 20 *μ*l volumes under the following conditions: 95°C for 10 min; then 40 cycles of 95°C for 30 s, 56°C for 30 s, and 72°C for 30 s; and finally 7 min at 72°C. The PCR product lengths for methylated and unmethylated *RECK* are 201 and 205 bp. CpGenome Universal Methylated DNA (Serologicals, Atlanta, Georgia, USA) and normal human blood DNA was used as positive control for methylated and unmethylated status. Water blank was used as a negative control. Positive and negative controls worked appropriately in each round of PCR. All assays were performed in triplicate.

### Reverse transcription–PCR and quantitative real-time PCR

Total RNA was isolated from 10^5^ to 10^6^ cultured cells using a Trizol reagent kit (Invitrogen, Carlsbad, CA, USA). cDNA was synthesised from 1 *μ*g of total RNA using MMLV Reverse Transcriptase (Invitrogen) with random hexamers. *RECK* cDNA was amplified by PCR using the sense primer 5′-GCAGGGGAAGTTGGTTGTTA-3′ and antisense primer 5′-TGCCAGCAAAACAAGAACAG-3′. Reactions were performed in 20 *μ*l volumes under the following conditions: 95°C for 10 min; then 35 cycles of 95°C for 30 s, 60°C for 30 s, and 72°C for 30 s; and finally 7 min at 72°C. Glyceraldehyde-3-phosphate dehydrogenase (GAPDH) was used as an internal control to estimate the efficiency of the cDNA synthesis in each cell line with forward primer: 5′-AGCATCTACACCTGAGGACAAGAC-3′; reverse primer: 5′-TTTTGCTCTTAACCACGTTTATTGA-3′. The integrated optical density of each band was quantified by densitometry. The relative levels of RECK mRNA were normalised against the GAPDH.

Quantitative real-time PCR assay was performed using SYBR®Premix Ex Taq™ in Thermal Cycler Dice® (Takara, Tokyo, Japan). The cycling conditions were initial denaturation at 95°C for 10 s, and 40 cycles at 95°C for 5 s, 60°C for 30 s according to the SYBR®Premix Ex Taq (Perfect Real Time) protocol. Second derivative maximum method was used to calculate the *C*_t_ (threshold cycle) value and standard curve method was used for relative quantification analysis. The *C*_t_ value of RECK was normalised by the *C*_t_ of GAPDH in the same sample. Each reaction was run in triplicate.

### Gelatin zymography

Cancer cell lines were seeded in bio-coat culture disks (Becton Dickinson Labware, Bedford, MA, USA) for 6 days. Fresh medium without FBS was replaced 24 h before analysis. MMP-2 and MMP-9 enzymatic activity in cancer cell lines was determined by sodium dodecyl sulphate (SDS) polyacrylamide gel electrophoresis (PAGE) gelatin zymography as described in our previous study (Kato *et al*, 2005). In brief, the gelatinolytic activity was measured in the presence of both proforms and activated forms of the MMP-2 and MMP-9. The cell extract was diluted in the ratio of 1 : 1 with sample buffer (TEFCO, Tokyo, Japan) and left at room temperature for 10 min. The cell extracts were applied to SDS–PAGE using a 10% gel containing 0.1% gelatin. After electrophoresis the gel was rinsed with renaturing buffer (TEFCO) for 30 min, and the gel was incubated in developing buffer (TEFCO) for 48 h at 37°C. After incubation the gel was stained with 0.05% Coomassie brilliant blue R250 (Wako Pure Chemical Industries, Osaka, Japan). The MMPs were detected as transparent bands on the blue background of the Coomassie blue stained slab gel.

### Collagen gel invasion model

Collagen gels were used as matrices for cancer cell invasion and prepared according to the method of Hase *et al* (2006) with a little adjustment. Type I collagen (Nippon Meat Packers Inc., Osaka, Japan) was mixed with medium and × 10 PBS at a ratio of 1 : 1 : 8 and air-vacuumed for 30 min before incubated in 5% CO_2_ and 37°C until gelling was completed. Cancer cells (3 × 10^5^) from each cell line were seeded on the collagen gels in medium with and without EGCG or 5-aza-dC. The cancer cells were maintained at 5% CO_2_ atmosphere and 37°C for 7 and 14 days. The collagen gel was collected and fixed in 10% formalin, embedded in paraffin, stained with haematoxylin and eosin and examined for cancer cell invasion.

### Assays for cell invasion and migration

Cell invasion and immigration assays were used to assess the formation of invasive foci and the depth of cell invasion into the collagen matrix previously described by Hotary *et al* (2000) and Liebersbach and Sanderson (1994). Six days after the addition of cells to gels, invasive foci were counted in randomly selected fields at × 200 magnification on phase-contrast microscope. The depth of cell invasion was determined by measuring the distance from the top of the gel to the leading front of migrating cells. The leading front distance was defined as the point at which two of the most distantly migrating cancer cells were simultaneously in focus in one field under × 200 magnification. Measurements were made using the calibrated micrometer of a Nikon inverted microscope. Measurements were taken in five fields within each well, and the mean distance was determined.

To measure cell proliferation rates, 8 × 10^4^ cells were placed into each culture plate and cultured for 3, 5, 7 days in medium without and with 50 *μ*M EGCG and 8.7 *μ*M 5-aza-dC. At each time point, the cells were harvested by trypsinisation and counted using a hemacytometer.

Cell migration ability was assessed by seeding 2–5 × 10^5^ cells atop collagen gels with surfaces partially covered by glass coverslips (1 × 2 cm; Matsunami Glass Ind, Ltd., Osaka, Japan). When the cells reach confluence, the glass fragments were removed, leaving a cell-free area on the gel. At this time, medium was added to all cultures in the absence or presence of EGCG and 5-aza-dC. The distances migrated across the gels were observed 24 h later using an inverted microscope.

### Statistical analysis

Differences between treatment and control groups were assessed by analysis of variance with *post hoc* test (Dunnett's test). Statistical analyses on the invasive foci and depth of invasion of the cancer cell lines were performed using the Student's *t*-test. The results were considered statistically significant at *P*<0.05.

## Results

### Methylation status and expression of the *RECK* gene in OSCC cell lines

**Figure 1:**
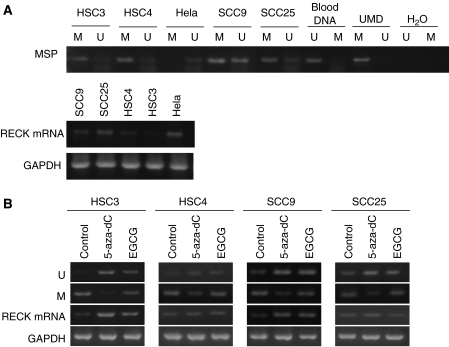
Methylation status and mRNA expression levels of the *RECK* gene. (**A**) Methylation analysis of the *RECK* promoter and mRNA expression in OSCC and HeLa cell lines. M, Methylation-specific band; U, Unmethylation-specific band; Blood DNA, normal human blood DNA as positive control for unmethylated status; UMD, Universal methylated DNA as positive control for methylated status; H20, Negative control. (**B**) Alterations of methylation status and mRNA expression levels of the *RECK* gene in OSCC cell lines after treatment with 50 *μ*M EGCG or 8.7 *μ*M 5-aza-dC for 6 days. GAPDH was used as an internal control.

A hypermethylated *RECK* promoter was observed in all four OSCC cell lines (100%) by MSP. SCC9 and SCC25 cell lines contained both unmethylated and methylated promoters whereas HSC4 and HSC3 cell lines had strongly methylated promoter and faintly unmethylated promotor. Methylation of *RECK* gene was not detected in HeLa cancer cell line whereas its unmethylation-specific band appeared as a weak band (Figure 1A). The expression levels of *RECK* mRNA in 4 human oral cancer cell lines and HeLa were examined by RT–PCR. The results showed that the HSC3 and HSC4 cell lines expressed very low *RECK* mRNA levels. SCC9 and SCC25 cells had downregulated expression whereas HeLa produced a normal amount of *RECK* mRNA (Figure 1A).

### Reversal of hypermethylation status and enhanced expression of RECK gene in OSCC cell lines by EGCG

To evaluate whether methylation status of *RECK* promoter is associated with transcriptional downregulation of *RECK* gene in the OSCC cell lines, demethylation treatment by 5-aza-dC and EGCG were performed on the OSCC cell lines. In all cancer cell lines, the *RECK* gene had hypermethylation status with the low level of the respective mRNA expression. The appearance of unmethylation-specific bands of *RECK* gene in all four cancer cell lines became more intense after treatment with 50 *μ*M EGCG or 8.7 *μ*M 5-aza-dC for 6 days. Treatment of SCC9 and HSC3 with EGCG and 5-aza-dC for 6 days also enhanced the transcription of *RECK* mRNA whereas *RECK* mRNA level was not significantly altered in HSC4 and SCC25 after treatment with EGCG (Figure 1B).

**Figure 2:**
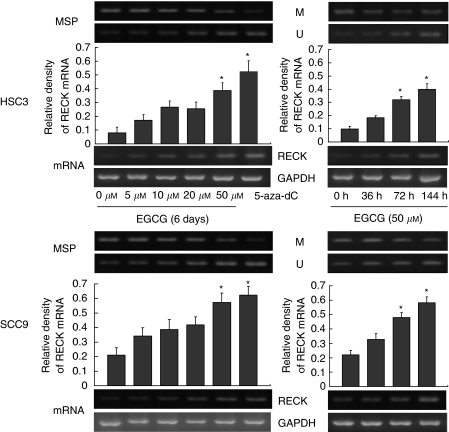
Dose-dependent and time course study. SCC9 and HSC3 cells were treated with 5, 10, 20, or 50 *μ*M EGCG or 8.7 *μ*M 5-aza-dC for 6 days. Cell lines were treated for 32, 72, and 144 h with 50 *μ*M of EGCG. The relative levels of RECK mRNA were normalised to the GAPDH. Band density was determined with densitometry. The bars represent mean±s.d. (*n*=3). ^*^P<0.01when compared with control.

We examined the time-dependent and dose-dependent effects of EGCG in SCC9 and HSC3 cell lines. After treating cells with 5, 10, 20, or 50 *μ*M of EGCG for 6 days, the methylation-specific bands of this gene still existed but in a weak appearance. The unmethylation-specific bands of *RECK* gene, however, appeared to be enhanced after treatment with 20 or 50 *μ*M of EGCG for 6 days. Corresponding to the appearance of the unmethylation-specific band was the increase of expression of *RECK* mRNA by conventional RT–PCR. The unmethylation-specific bands and mRNA expression were significantly stronger with 50 *μ*M of EGCG in comparison with those treated with 20 *μ*M EGCG or lower concentrations (*P*<0.01). The reversal of hypermethylation and increase of expression of *RECK* by EGCG were almost similar to that produced by the classical DNMT inhibitor, 5-aza-dC. After treating the cells with 50 *μ*M of EGCG for 36, 72, and 144 h, strong unmethylation-specific bands of the *RECK* gene began to appear at 72 h. The higher level of mRNA expression of *RECK* was also observed at 72 and 144 h (*P*<0.01) (Figure 2).

**Figure 3:**
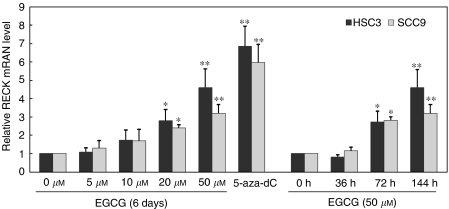
Relative mRNA expression levels of *RECK* gene by quantitative real-time PCR in HSC3 and SCC9 cell lines. EGCG enhanced RECK mRNA expression in a dose- and time-dependent manner. Expression levels were normalised to the human GAPDH control gene, an endogenous control. All expression levels are shown relative to the untreated sample. The bars represent mean±s.d. (*n*=3). ^*^P<0.01; ^**^P<0.001when compared with control.

To confirm the effect of EGCG on *RECK* expression, real-time quantitative PCR was performed to determine the mRNA expression level of *RECK* gene in HSC3 and SCC9 cell lines after treatment with different concentrations of EGCG. The results showed that the relative amount of mRNA expression of *RECK* was increased in a dose- and time-dependent manner with significant effect at 20 and 50 *μ*M EGCG (*P*<0.01 and *P*<0.001) and at 72 and 144 h (*P*<0.01 and *P*<0.001). This result was generally consistent with those from general reverse transcription–PCR, however, with a significant effect at lower concentration of EGCG (20 *μ*M) (Figure 3).

### Downregulation of MMPs by EGCG

**Figure 4:**
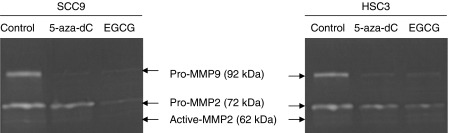
Gelatin zymography analysis. Clear zones of proform and active MMP-2 (72 and 62 kDa) and relatively weaker MMP-9 bands (92 kDa proform and 82 kDa active form) that attenuated following the 50 *μ*M EGCG or 5-aza-dC 8.7 *μ*M treatment for 6 days in SCC9 and HSC3 cells.

Gelatin zymography results showed that MMP-2 and MMP-9 expression and activity were suppressed in SCC9 and HSC3 cells treated with EGCG and 5-aza-dC for 6 days (Figure 4). Culture with EGCG or 5-aza-dC decreased total MMP-2 levels and downregulated the activation of proMMP-2. Although proMMP-9 was detectable, active forms of MMP-9 were not readily observed in this experiment. Treatment with 50 *μ*M EGCG did not cause downregulation in MMP-9 activity but MMP-2 in HSC4 and SCC25 cancer cell line (data not shown).

### Inhibition of cancer invasion in collagen matrices by EGCG

**Figure 5:**
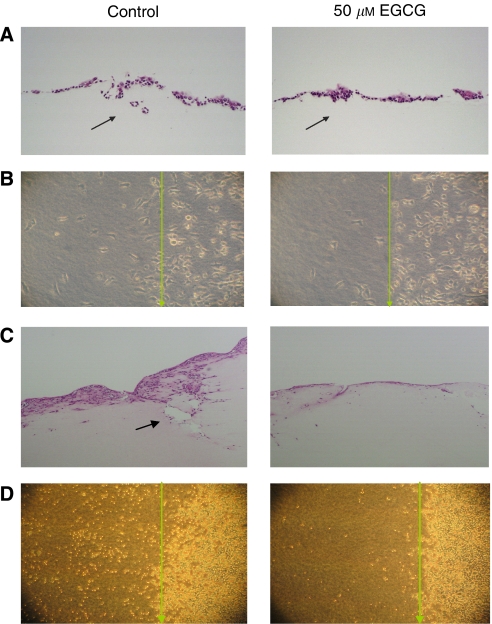
Collagen gel invasion model. Treatment of EGCG for 6 days inhibits cancer invasion in collagen model in HSC3 cells. (**A**) The collagen gel was collected and fixed in 10% formalin, embedded in paraffin, stained with haematoxylin and eosin and examined for cancer cell invasion. Treatment with EGCG significantly inhibits invasion in collagen model for 6 days (Arrow is showing an invasive foci). (**B**) Cell migration ability was suppressed in the presence of EGCG for 6 days. Cells atop collagen gels with surfaces were partially covered by glass coverslips (1 × 2 cm). When the cells reach confluence, the glass fragments were removed, leaving a cell-free area on the gel. The distances migrated across the gels were observed 24 h later using inverted microscope. (**C**) Inhibition of cell invasion by EGCG in HSC3 cells for 14 days. (**D**) The ability of cancer cells to migrate from the edge towards centre of the uncovered gel in HSC3 cells cultured with EGCG were decreased as compared to control on day 14 (after 24 h of removing the cover slips) ( × 100 magnification). The arrows show the margin of the uncovered area on the gel.

When cultured atop collagen gels, SCC9, SCC25, and HeLa cells formed confluent monolayers that remained confined to the surface of the underlying gel for the entire culture period regardless of the presence of EGCG or 5-aza-dC. We also did not observe the significant invasion in the SCC9 and SCC25 cells. However, treatment with EGCG significantly inhibits cancer-invasive ability in collagen model in HSC4 and HSC3 cancer cell lines (Figure 5A–D). During 7 days treatment with EGCG, HSC3 and HSC4 cells proliferated at a lower rate compared with those of control or 5-aza-dC treated cells. Although treatment with 50 *μ*M EGCG caused some damaged cells with vesicles, signs of toxicity were not apparent at lower doses.

**Figure 6:**
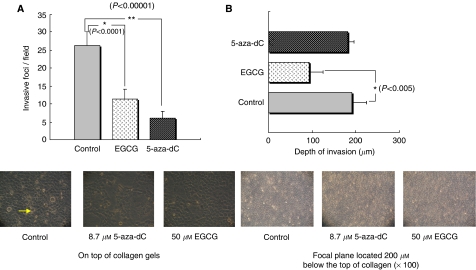
Invasive foci formation and invasion depth of cancer cells were inhibited by EGCG. (**A**) On top of collagen gels ( × 200 magnification) EGCG or 5-aza-dC significantly blocked cancer invasion in HSC3 cells by decreasing the mean number of invasive foci/field by *t*-test. Results are shown as the mean number of invasive foci ±1 s.d. in five randomly selected fields (6 days). (**B**) Focal plane located 200 *μ*m below the top of collagen ( × 100 magnification). 6 days after addition of HSC3 cells to gels, the depths of cell invasion of cells that had invaded the gel were significantly blocked in cells treated with EGCG. Similar results were observed with HSC4 cell line (not shown).

Our results indicated that treatment with 50 *μ*M EGCG significantly inhibited cancer invasion in a three-dimensional collagen model. Furthermore, EGCG or 5-aza-dC significantly blocked cancer invasion in HSC3 cells by decreasing the mean number of invasive foci/field (*P*<0.0001 and *P*<0.00001, respectively). Results are shown as the mean number of invasive foci ±1 s.d. in five randomly selected fields in HSC3 cells treated with 5-aza-dC, EGCG and control (6±2 foci, 11.4±2.7 foci, and 26.4±3.8 foci, respectively). Interestingly, regarding cell migration and depth of invasion, EGCG significantly inhibited cells to invade deeply into the collagen gel compared with control (93±32.3 *vs* 191.2±33.7 *μ*m, *P*<0.005) as well as suppressed the cells migrating across the gels surface (Figure 5B, D and 6A, B). Similar effects were also observed in HSC4 cell line (Table 1).

In the presence of EGCG, HSC3 and HSC4 cells cultured atop collagen gels for 6 days displayed widespread but shallow foci of invasion. In contrast, control cells (EGCG-free medium) invaded and generated large and deep pits extending well in the collagen matrix. After 14 days, HSC3 cells cultured in the absence of EGCG formed an aggressive invasion and proliferation (with stratified 5–6 cell layers) whereas invasion of HSC3 cells treated with EGCG was markedly inhibited and only generated pits underlying collagen gels (Figure 5C and D).

## Discussion

In the present study, we report that EGCG, a major component of green tea, may enhance *RECK* expression by reversal of hypermethylation of *RECK* promoter and inhibit MMP activities as well as cancer cell invasion in OSCC cell lines. Recent studies have also shown that *RECK* methylation is associated with increase of metastasis and invasion in human cancers (Chang *et al*, 2006, 2007; Cho *et al*, 2007).

Our findings implied that the hypermethylation of *RECK* is associated with a low level of mRNA expression in oral squamous cell carcinoma cell lines. Of these, SCC9 and SCC25 cell lines were partially methylated whereas HSC4 or HSC3 cells were almost completely methylated. The results showed that treatment of oral cancer cells with EGCG partially reversed the hypermethylation status of the *RECK* gene and significantly enhanced the expression level of *RECK* mRNA. EGCG has been reported to reverse hypermethylation and reactivate several tumour suppressor genes in human oesophageal squamous cell carcinoma cell lines at doses from 20 *μ*M (Fang *et al*, 2003) by nested two-stage MSP. However, present oral cancer cell lines appear to be less susceptible to a demethylation effect by EGCG. Additional work is needed to determine whether different genes respond similarly or differently to the EGCG treatment in different cell lines under various treatment conditions.

EGCG has been shown to affect MMP activity both directly and indirectly, in particular MMP-2 at relatively low doses (10–20 *μ*M). EGCG has been reported to inhibit activating protein-1 (AP-1) that regulates MMP expression. In another way, EGCG could also inhibit the proMMP-2 protein secretion by perturbing the intracellular vesicular trafficking (Oh *et al*, 2004; Khan *et al*, 2006). However, our data demonstrate that OSCC cell lines-derived MMP-2 and MMP-9 were inhibited at 50 *μ*M of EGCG treatment. Previous studies showed that restored expression of *RECK* in malignant cells resulted in suppression of invasive activity with concomitant decrease in the secretion of MMPs (Takahashi *et al*, 1998; Okabe *et al*, 1999; Liu *et al*, 2003; Noda *et al*, 2003). Treatment with EGCG, HSC3 and HSC4 cells cultured on top of collagen gels produced widespread but shallow foci of invasion whereas control cells (EGCG-free medium) invaded and generated large and deep pits extending well in the collagen matrix. This could be because of the ability to regulate MMPs by *RECK* restored expression. Earlier study has shown that stimulated *RECK* expression significantly caused downregulation of MMP-2 and MMP-9 activities (Takahashi *et al*, 1998; Liu *et al*, 2003; Oh *et al*, 2004). In experimental systems, cellular invasion is reduced by the presence of endogenous tissue inhibitors of MMPs (*TIMPs*) and *RECK*. Recently, *TIMP-2* has been reported to enhance expression of *RECK* through Rap1 signalling resulting in an indirect, time-dependent inhibition of cell migration (Oh *et al*, 2004). Our findings suggest that EGCG and 5-aza-dC may act simultaneously through different target proteins such as TIMPs and RECK to suppress invasion. Further study is needed to define the extent of contribution of *RECK* on MMPs to the suppression of cell-invasive behaviours.

The results support the conclusion that EGCG plays a crucial role in inhibiting cell invasion of a collagen model. EGCG significantly suppressed cancer-invasive ability in oral cancer cell lines by decreasing the number of invasive foci and depth of invasion as strong as classical DNMT inhibitor, 5-aza-dC. Oral administration of green tea has been reported to inhibit tumour genesis in different organs and multiple mechanisms may be involved (Jung and Ellis, 2000; Khan *et al*, 2006; Chiang *et al*, 2006; Okabe *et al*, 1999). Importantly, these effects of EGCG have been shown to be selective for cancer cells, as normal cells were not affected by this treatment (Khan *et al*, 2006).

The present study indicated that the effective dose of EGCG in inhibiting cancer invasion and migration is at 50 *μ*M. At a concentration of 20 *μ*M or lower, EGCG did not significantly affect cancer invasion or migration of the cells. It is achievable in the oral cavity (in saliva) after drinking green tea and perhaps in the stomach, esophagus and the intestines where there is direct contact between EGCG and the epithelial cells (Fang *et al*, 2003). This effective concentration is variable in different organs and the DNMT inhibition would depend on the systemic bioactivities and the bioavailability of EGCG in a particular organ site. Inhibition of DMNT is expected to prevent hypermethylation; however, severe inhibition of DNMT activity may cause DNA hypomethylation, genomic instability and other disorders (Szyf *et al*, 2004; Wilson *et al*, 2007). The effects of EGCG *in vitro* have been obtained with relatively high concentrations than observed in the plasma or tissues of animals or in human plasma after the administration of green tea or EGCG (Masuda *et al*, 2001; Khan *et al*, 2006). Therefore, it is not clear whether the activities observed with high EGCG concentrations in cell lines can be observed *in vivo*. More comprehensive studies in animal models and humans are needed to determine the optimal doses and side effects of EGCG.

In conclusion, our findings raise the possibility that EGCG could inhibits cancer cell invasion through reversal of hypermethylation status of *RECK* and downregulation of MMP-2 and MMP-9. Our data together with earlier studies indicate that EGCG as a natural demethylating agent could be a promising therapeutic strategy for the development of combination chemopreventive/chemotherapeutic approaches in oral cancer treatment. Additional investigations are required to fully elucidate the molecular mechanisms by which green tea constituents, and EGCG in particular, inhibit tumour invasion and metastasis.

## Figures and Tables

**Table 1:** Mean number of invasive foci per field and depth of invasion in HSC3 and HSC4 cancer cell lines after treatment with 50 *μ*M EGCG or 8.7 *μ*M 5-aza-dC for 6 days

	**Number of invasive foci per field mean±s.d.**			**Depth of invasion mean±s.d. (μm)**		
**Cell line**	**Control**	**50 *μ*M EGCG**	**8.7 *μ*M 5-aza-dC**	**Control**	**50 *μ*M EGCG**	**8.7 *μ*M 5-aza-dC**
HSC3	26.4±3.8	11.4±2.7^a^	6.0±2.0^b^	191.0±33.7	93.0±32.3^c^	182.4±13.2
HSC4	17.6±2.9	10.2±0.8^d^	10.6±3.6^c^	234.6±27.9	180.4±16.1^a^	206.4±25.9

a *P*<0.00001;

b *P*<0.0001;

c *P*<0.001;

d *P*<0.005 when compared with control by the Student's *t*-test.
